# Identification and validation of a novel cuproptosis-related genes signature associated with prognosis, clinical implications and immunotherapy of hepatocellular carcinoma

**DOI:** 10.3389/fphar.2023.1088993

**Published:** 2023-02-09

**Authors:** Fengjiao He, Puhua Zeng, Sijing Ma, Ximing Yang, Huan Liu, Qiong Liu, Yangying Zhou, Hong Zhu

**Affiliations:** ^1^ Department of Oncology, Xiangya Hospital, Central South University, Changsha, China; ^2^ National Clinical Research Center for Geriatric Disorders, Xiangya Hospital, Central South University, Changsha, China; ^3^ Hunan Academy of Traditional Chinese Medicine Affiliated Hospital, Changsha, China; ^4^ Medical School, Hunan University of Chinese Medicine, Changsha, China

**Keywords:** hepatocellular carcinoma, cuproptosis-related genes, tumor microenvironment, drug sensitivity, prognosis model

## Abstract

**Background:** Cuproptosis is a novel type of regulated cell death and is reported to promote tumor occurrence and progression. However, whether a cuproptosis-related signature has an impact on hepatocellular carcinoma (HCC) is still unclear.

**Materials and methods:** We analyzed the transcriptome data of HCC from The Cancer Genome Atlas (TCGA) and International Cancer Genome Consortium (ICGC) database, and searched for tumor types with different cuproptosis patterns through consistent clustering of cuproptosis genes. We then constructed a Cuproptosis-Related Genes (CRGs)-based risk signature through LASSO COX regression, and further analyzed its impact on the prognosis, clinical characteristics, immune cell infiltration, and drug sensitivity of HCC.

**Results:** We identified the expression changes of 10 cuproptosis-related genes in HCC, and all the patients can be divided into two subtypes with different prognosis by applying the consensus clustering algorithm. We then constructed a cuproptosis-related risk signature and identified five CRGs, which were highly correlated with prognosis and representative of this gene set, namely *G6PD*, *PRR11*, *KIF20A*, *EZH2*, and *CDCA8*. Patients in the low CRGs signature group had a favorable prognosis. We further validated the CRGs signature in ICGC cohorts and got consistent results. Besides, we also discovered that the CRGs signature was significantly associated with a variety of clinical characteristics, different immune landscapes and drug sensitivity. Moreover, we explored that the high CRGs signature group was more sensitive to immunotherapy.

**Conclusion:** Our integrative analysis demonstrated the potential molecular signature and clinical applications of CRGs in HCC. The model based on CRGs can precisely predict the survival outcomes of HCC, and help better guide risk stratification and treatment strategy for HCC patients.

## 1 Introduction

Hepatocellular Carcinoma (HCC) is one of the most common malignant tumors, and ranks the sixth most common and third mortality in all tumors worldwide by the World Health Organization (WHO) (Siegel et al., 2022)- (Ferlay et al., 2021). Although early-stage HCC can be cured by surgical treatment, enormous challenges remain in the treatment of advanced HCC, resulting in unfavorable prognosis, significant financial cost and high disease burden (Bandmann et al., 2015). Given the high morbidity and mortality of HCC, there is an urgent need to develop more effective prognostic models, and explore reliable prognostic factors, which is crucial for optimal individualized management and treatment.

Copper is an essential cofactor for all organisms, but copper is toxic if concentrations exceed a threshold maintained by evolutionarily conserved homeostatic mechanisms. However, how excess copper induces cell death is not known. The Broad institute currently uncovers a novel cell death mechanism, cuproptosis (Tsvetkov et al., 2022), which is distinct from the known apoptosis, necrosis, autophagy and iron death.

Cuproptosis is a form of copper-dependent and mitochondrial respiration-dependent, regulated cell death. Cuproptosis occurs by direct binding of copper to lipoylated components of the tricarboxylic acid (TCA) cycle (Beaino et al., 2014)- (Hatori et al., 2016), resulting in aberrant aggregation of lipoylated proteins and loss of ferroptosis proteins, leading to cell death by proteotoxic stress. Copper ions are involved in cell death as are iron ions, while the study from the Broad Institute demonstrates strategies to combat disease by pharmacologically inhibiting mitochondrial respiration (Tsvetkov et al., 2022). In addition, cancer cells are actively respiring and contain large amounts of lipoylated mitochondrial proteins. Copper ionophores could be used to destroy cancer cells, which opens up a new therapeutic direction for cancer. However, the metabolism of copper in liver diseases and the occurrence and development of HCC is still poorly understood. In the research stage. The evidence by Siddiqui et al., demonstrated that copper oxide nanoparticles have dose-dependent cytotoxicity and apoptotic effects on HepG2 cells (Siddiqui et al., 2013). Besides, copper contents were closely associated with liver cirrhosis and HCC, and serum levels of copper, like ceruloplasmin, may be used as a marker for the detection of HCC (Zhang et al., 1994). Recently, as reported, cuproptosis-related signature and the lncRNA profile linked with cuproptosis may bring new insights into the molecular pathways of the formation and progression of cancers, which were helpful to predict the prognosis and guiding treatment of cancer patients (Zhen et al., 2022)- (Zhang et al., 2022).

Emerging evidence also suggests crosstalk between curoprotosis and the tumor immune microenvironment (TME) (Lv et al., 2022)- (Li et al., 2022a). The tumor microenvironment plays a crucial role in cancer development and clinical outcomes (Wu and Dai, 2017). The TME includes cancer cells, immune cells, endothelial cells, inflammatory cells and fibroblasts, as well as extracellular components (growth factors, hormones, cytokines, etc.). Within the TME, interactions between cancer cells and immune cells regulate all links of tumor development, and tumor-infiltrating immune cells (TIICs) can also influence cancer progression (Lee and Cheah, 2019)- (Zhou et al., 2022a). Despite recent advances in immunotherapy for HCC, the prognosis of HCC remains heterogeneous, which suggests that the close connection between curoprotosis and the tumor immune microenvironment may play a crucial role in the development and progression of HCC. However, the role of cuprotosis-mediated gene patterns in HCC is unclear.

In this study, we comprehensively investigate the molecular alterations and clinical relevance of cuproptosis-related genes (CRGs) in HCC. We then constructed a cuproptosis-related risk signature and identified five CRGs for predicting survival outcomes and characterizing the immune landscape of HCC. Additionally, combined with clinicopathological features and treatment efficacy, the CRGs signature demonstrated great potential for precision and personalized therapy of HCC.

## 2 Materials and methods

### 2.1 Data download and preprocessing

Based on R package The Cancer Genome Atlas (TCGA) biolinks v1.16.0, the expression profile data (FPKM), genomic data (SNV and CNV) and clinical data of HCC were downloaded. Survival data were used from 2018 collated data (Liu et al., 2018). The TCGA HCC dataset (https://cancergenome.nih.gov/, version 27.0-fix, released on 9 November 2020) as training cohort, which included 269 HCC tumor samples and 50 tumor-adjacent normal tissues. In the meantime, the Liver Cancer-RIKEN-JP (LIRI-JP) of HCC transcriptome data (FPKM) and clinical survival data in the (International Cancer Genome Consortium) ICGC database (https://dcc.icgc.org/projects/LIRI-JP, version Release_28, processed on 27 March 2019) was used for the validation cohort, which contained 232 HCC cases. Above data, genes were removed when multiple ENSEMBL Identity Documents (ID) were encountered corresponding to the same SYMBOL. The batch effect between different datasets was corrected using the “sva” package of R software by adopting the “combat” algorithm. In addition, we filtered the genes that were expressed in less than 50% of the samples.

### 2.2 Difference analysis

Gene expression differences were calculated using DESeq2 through count expression profiles, and genes with an absolute value of Log2 Foldchange >1 and adjusted *p* values less than 0.05 were selected as differential genes. Multiple testing correction is based on the FDR method. Differential gene volcano plots were drawn by ggplot2 (3.3.6) and ggrepel (0.9.1) R packages, and significant cuproptosis-related genes were marked. The expression heatmap of Cuproptosis-related genes in HCC and normal tissues were plotted by the R package pheatmap (1.0.12).

### 2.3 Comparison of cuproptosis-related genes under different clinical

Based on the FPKM expression dataset of TCGA HCC and clinical feature data, we stratified the samples by TCGA molecular classification, alpha-fetoprotein value, bilirubin albumin maximum, fibrosis, grade, stage, age, gender, BMI, etc. We then calculated the expression differences of cuproptosis-related genes between groups by the Wilcoxon rank sum test. Boxplots were drawn using ggpubr (0.4.0) heatmaps were drawn using pheatmap (1.0.2).

### 2.4 Construction of protein interaction network

A PPI network was constructed based on ten cuproptosis-related genes using STRING (http://www.string-db.org/) (Szklarczyk et al., 2021), and Gene Ontology (GO) functional enrichment analysis was performed.

### 2.5 Gene correlation analysis

We extracted the expression values of cuproptosis genes or cuproptosis genes to immune checkpoints from the TCGA HCC FPKM data. We performed logarithmic transformation on the gene expression values, and calculated the correlation between the expression of the two genes by Pearson correlation analysis.

### 2.6 Identification of cuproptosis-associated tumor subtypes

Based on the TCGA dataset, we identified different subtypes based on the expression profile data of 10 cuproptosis-related genes, applying non-negative matrix decomposition and unsupervised consensus clustering analysis. We used the consensus cluster plus (4.5.1.902) and Non-negative matrix factorization (NMF) (0.24.0) packages to operate, and the consensus clustering used three clustering distances: Spearman, Pearson, as well as Euclidean. The clustering method was K-means clustering with l000 replicates to guarantee the stability of the classification. We selected consensus clustering (Euclidean distance) to determine the tumor cuproptosis subtype based on the idea that the survival *p*-value was minimally separated.

### 2.7 Functional enrichment analysis

GO and Kyoto Encyclopedia of Genes and Genomes (KEGG)pathway enrichment analyses were performed based on significantly differentially expressed genes and the R package cluster profiler (4.2.2), and results with FDR corrected *p*-values less than 0.05 were selected and the top few pathways were displayed using bubble plots (Li et al., 2022b). Construction of the cuproptosis-associated signature based on the two identified subtypes of cuproptosis tumors. In the transcriptome data of TCGA HCC, all gene expression values were divided into two groups according to the median value. Univariate Cox regression analysis was performed using the R package survival (3.3–1). Genes with a *p*-value less than 0.05 were filtered out, and cuproptosis-associated genes were further constructed by the R package glmnet (4.1–4) Lasso Cox regression to remove redundant genes, according to the following formula signature.

### 2.8 Survival analysis

In the TCGA database and ICGC validation dataset, median grouping was performed based on the calculated cuproptosis score, and the impact on prognosis was assessed by constructing Kaplan-Meier curves using the survival (3.3–1) R package and the log-rank test. ROC curve was plotted using the R package timer0c (0.4), and Cox regression (R package survival 3.3–1)) was performed to calculate hazard ratios (HR) for scoring groups and clinical characteristics.

### 2.9 Gene mutation and copy number variation analysis

The single nucleotide variation and copy number variation data of HCC The genes with mutation frequencies greater than 5% in the high and low copper death signature groups were then displayed using oncoPrint through the R package ComplexHeatmap (version 2.10.0), and the chi-square test was used to determine whether there was a significant difference between the two groups. The GenVisR (1.26.0) package defines low copy number variation with copy number < 1, and copy number > 3 as high fold variation, showing the copy number variation of the high and low groups.

### 2.10 Immune cell infiltration calculation

Using the R package IBOR (0.99.9) based on ESTIMATE (Aran et al., 2015), Microenvironment Cell Populations-counter (MCP-counter) (Giraldo et al., 2016), XCELL (Aran et al., 2017) and CIBERSORT (Newman et al., 2015)immune cell infiltration algorithms, the score of each immune cell in the HCC sample was calculated. The Wilcoxon rank test was used to compare the different levels of cuproptosis signature between the two groups with immune cell infiltration.

### 2.11 Drug sensitivity prediction

Based on Genomics of Drug Sensitivity in Cancer (GDSC) (Yang et al., 2013), Cancer Cell Line Encyclopedia (CCLE) (Barretina et al., 2012) and Cancer Therapeutics Response Portal (CTRP) (Basu et al., 2013) drug databases, we extracted cancer cell line expression data, calculated the cuproptosis fraction of each cell line, and grouped them based on the median gene expression. We then combined the genes expression with the Area Under the Curve (AUC) and Half maximal inhibitory concentration (IC50) data of multiple drugs in cell lines, and use Spearman’s correlation to calculate the correlation with cuproptosis score, and further used the Wi1coxon test to compare the difference of AUC/IC50 between high and low cuproptosis groups in significantly related drugs.

### 2.12 Impact of immunotherapy response

Based on TCGA’s HCC transcriptional data, we used the Tumor Immune Dysfunction and Exclusion (TIDE) tool (http://tide. dfci.harvard. edu/) (Jiang et al., 2018) to predict the immunotherapy response of the samples and compared the difference in scores between the responder and non-responder groups. The Wilcoxon rank test was used for a statistical test and the difference in the proportion of response and non-response between the two groups with high and low cuproptosis scores was compared.

## 3 Results

### 3.1 The landscape of cuproptosis-related genes in HCC

**FIGURE 1:**
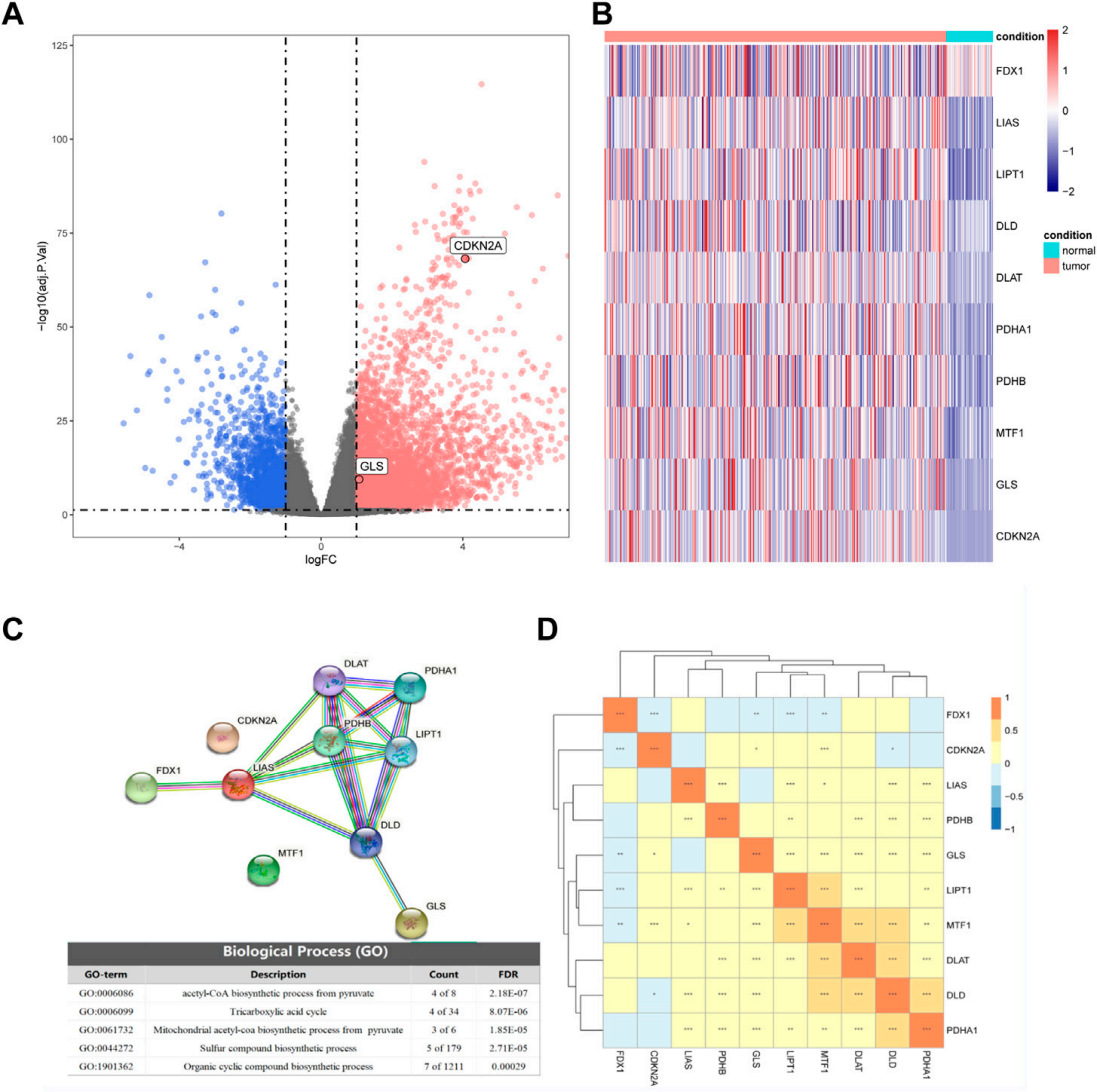
Differential expression of cuproptosis-related genes in hepatocellular carcinoma (HCC) and normal tissues. **(A)** Volcano plot of differential expression (blue represents downregulation in HCC, red represents upregulation in HCC, gray represents insignificant, and cuproptosis-related genes are marked in the figure). **(B)** The expression of 10 cuproptosis-related genes in HCC and normal tissues. **(C)** Partial results of protein-protein interactions (PPI) network map and gene ontology (GO) enrichment of 10 cuproptosis-related genes. **(D)** Heatmap of expression correlation of 10 cuproptosis-related genes in TCGA.

Based on the TCGA transcriptome dataset, we performed differential gene analysis between HCC tumor and adjacent normal tissues, and explored 6,031 differential genes, of which 1,503 were downregulated and 4,528 were upregulated Figure 1A). We then plotted the heatmap by using the R to scale the FPKM of gene expression (Z-score). Among these differential genes, we discovered that CDKN2A and GLS were significantly upregulated in HCC among all the cuproptosis-related genes, and these two genes might contribute to the development of HCC (Figure 1B).

By analyzing the expression correlations of the 10 cuproptosis genes, we found that LIAS, LIPT1, DLD, DLAT, PDHA1, PDHB, MTF1, GLS, and CDKN2A showed positive correlations with other genes, while FDX1 was negatively correlated with the expression of other genes (Fig 1D). The protein-protein interaction (PPI) network of GO enrichment analysis revealed that the CRGs participated in compound biosynthesis and energy metabolism (Figure 1C).

**FIGURE 2:**
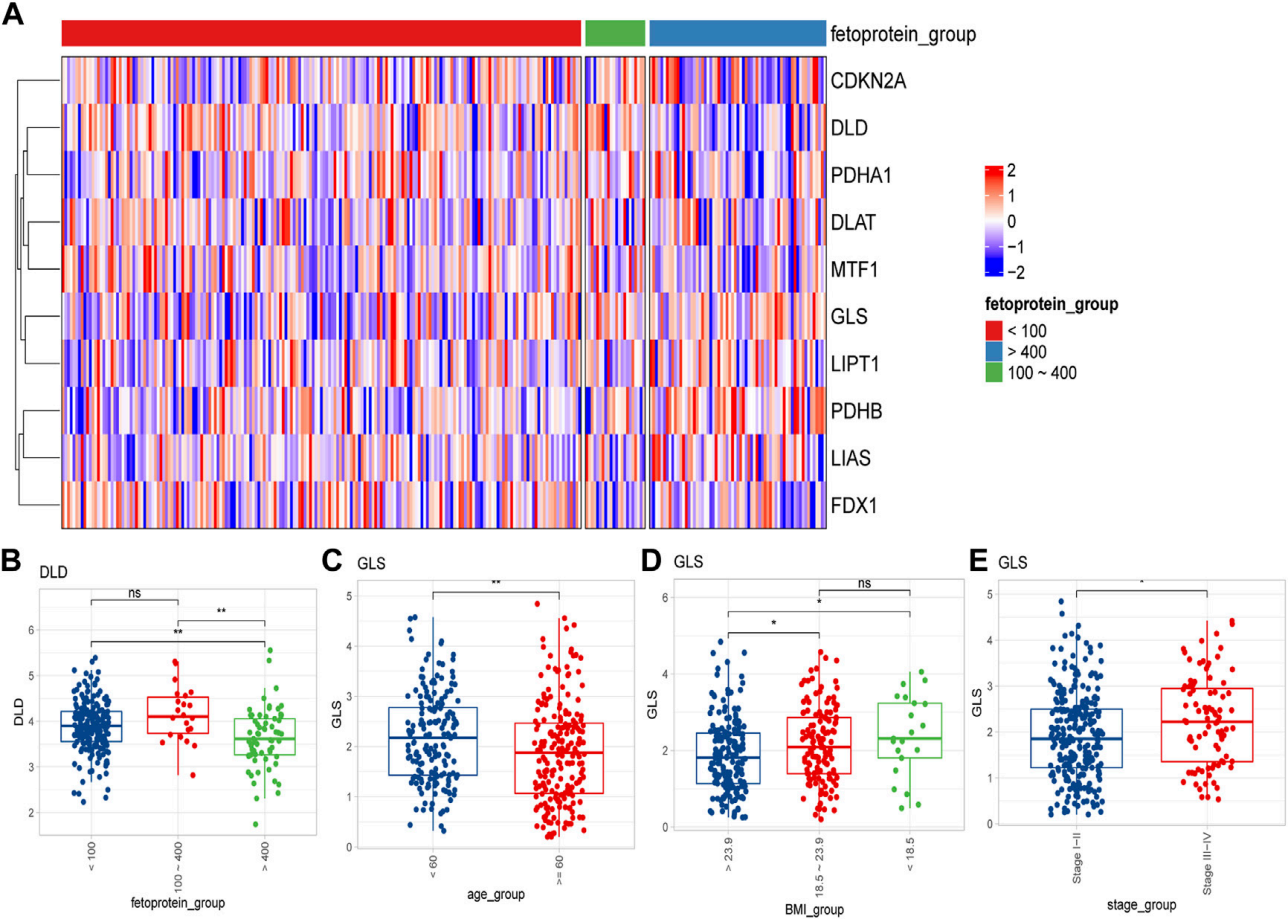
Differential expression of cuproptosis-related genes in different clinical feature groups. **(A)** Expression of 10 cuproptosis-related genes in different alpha-fetoprotein groups (<100 mg/dL, 100–400 mg/dL, >400 mg/dL); **(B)** *DLD* expression differences in different alpha-fetoprotein groups; **(C–E)** The difference of *GLS* expression in different age, BMI and different tumor stage groups (ns: *p* >0.05; *: *p* <0.05; **: *p* <0.01; ***: *p* <0.001).

We then compared the expression differences of CRGs among diverse clinical characteristics. We observed that different CRGs were differentially expressed in distinct signatures, such as *GLS* showing distinct expression differences in different age, Body Mass Index (BMI) subgroups, as well as different tumor stages (Figures 2A, C–E) Besides, *DLD* was significantly expressed at different α-fetoprotein levels (Figure 2B).

### 3.2 Identification and characterization of cuproptosis-related molecular subtypes in HCC

**FIGURE 3:**
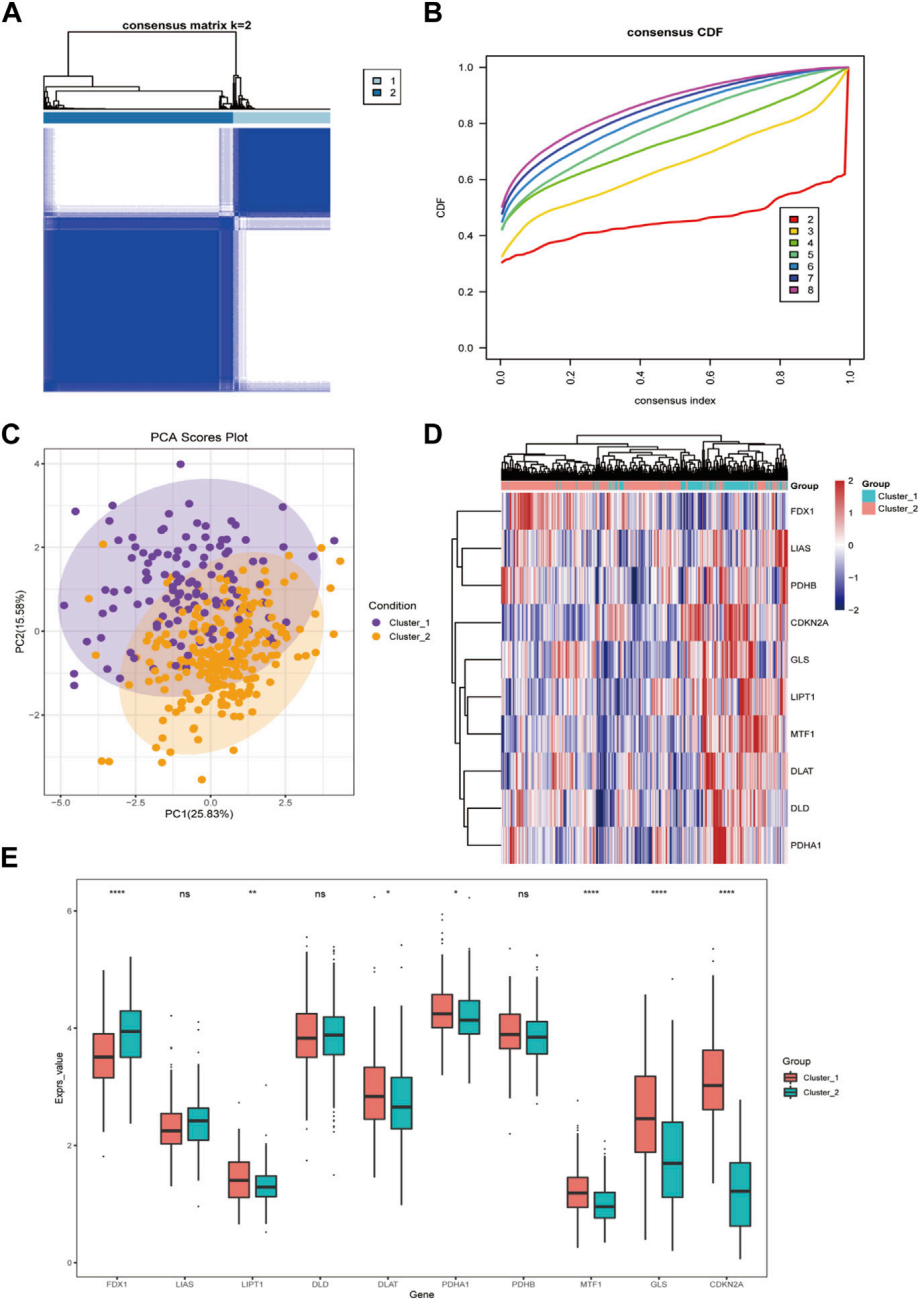
The cuproptosis-related genes divide hepatocellular carcinoma into two subtypes. **(A)** The sample squareness of the consistent clustering (number of classifications = 2); **(B)** The cumulative distribution map of the consistent clustering; **(C)** The principal component analysis graph of the two hepatocellular carcinoma subtypes; **(D)** The cuproptosis-related genes in the two categories Expression heatmap of hepatocellular carcinoma; **(E)** Boxplot of cuproptosis-related genes expression difference between two types of hepatocellular carcinoma (ns: *p* >0.05; *: *p* <0.05; **: *p* <0.01; ***: *p* <0.001; ****: *p* <0.0001).

Firstly, we applied a consensus clustering algorithm to categorize the HCC patients based on the expression of 10 CRGs. The consistency coefficient was evaluated to determine the optimal clustering number (k value), and the results demonstrated that *k* = 2 was the best choice for dividing the cohort into two subgroups (Figures 3A, B). Based on the principal component analysis (PCA), the HCC patients were well separated into two categories (Figure 3C). We then discovered that *FDX1*, *LIPT1*, *MTF1*, *GLS*, and *CDKN2A* was significantly differentially expressed between the two groups (Figures 3D, E).

**FIGURE 4:**
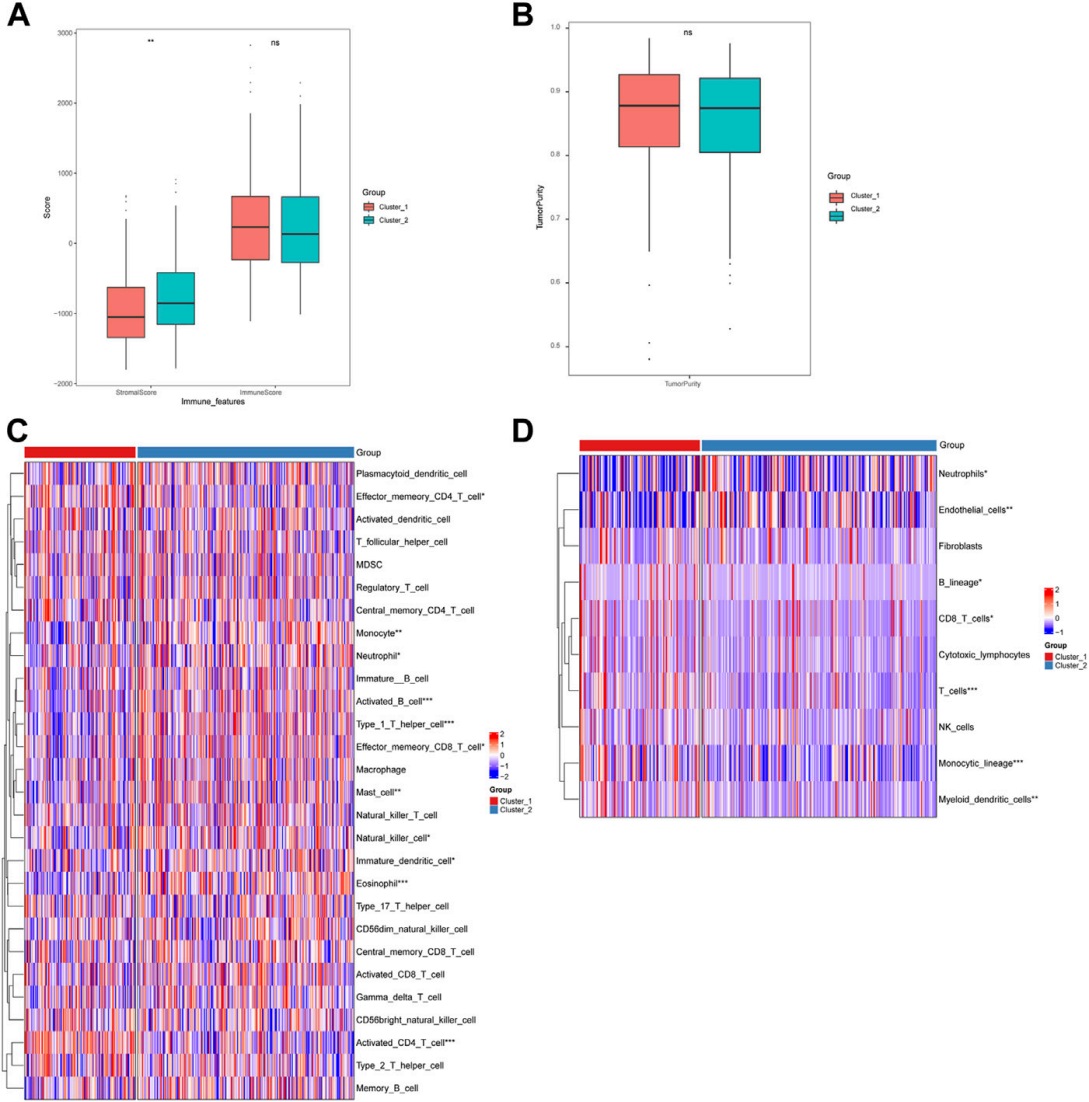
Differences in immune cell infiltration among hepatocellular carcinoma subtypes. **(A)** ESTIMATE algorithm calculates differences in stromal and immune scores between subtypes; **(B)** ESTIMATE algorithm calculates differences in tumor purity scores between subtypes; **(C)** GSVA-cell report algorithm calculates differences in immune cell infiltration between subtypes; **(D)** MCP-counter calculates differences in subtypes differences in immune cell infiltration (ns: *p* >0.05; *: *p* <0.05; **: *p* <0.01; ***: *p* <0.001; ****: *p* <0.0001).

Furthermore, we analyzed the immune cell infiltration scores by using CIBERSORT, GSVA-cellreport, ESTIMATE, and MCP-counter algorithms. We found that Cluster-2 scored higher for stromal cells, while no significant differences were observed for immune scores and tumor purity (Figures 4A, B). We also discovered significant differences between Cluster-1 and Cluster-2 for distinct immune cell infiltration, such as T cells, B cells and macrophages (Figures 4C, D).

### 3.3 Construction and validation of cuproptosis-related genes signature

**FIGURE 5:**
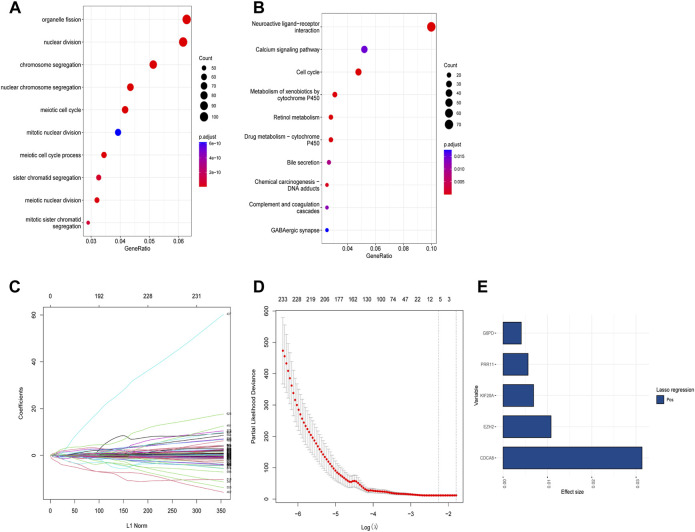
Functional analysis of different subtypes and the construction of cuproptosis-related genes (CRGs) signature in hepatocellular carcinoma. **(A)** Gene ontology (GO) enrichment analysis results in the two CRGs subtypes. **(B)** Kyoto Encyclopedia of Genes and Genomes (KEGG) enrichment analysis results in the two CRGs subtypes: **(C)** Lasso regression coefficients of each variable with L1 norm; **(D)** Lambda logarithm value in Lasso regression and the relationship with the error (the dotted line is the range that Lambda can choose); **(E)** The coefficient values of the Lasso regression screening variables.

**FIGURE 6:**
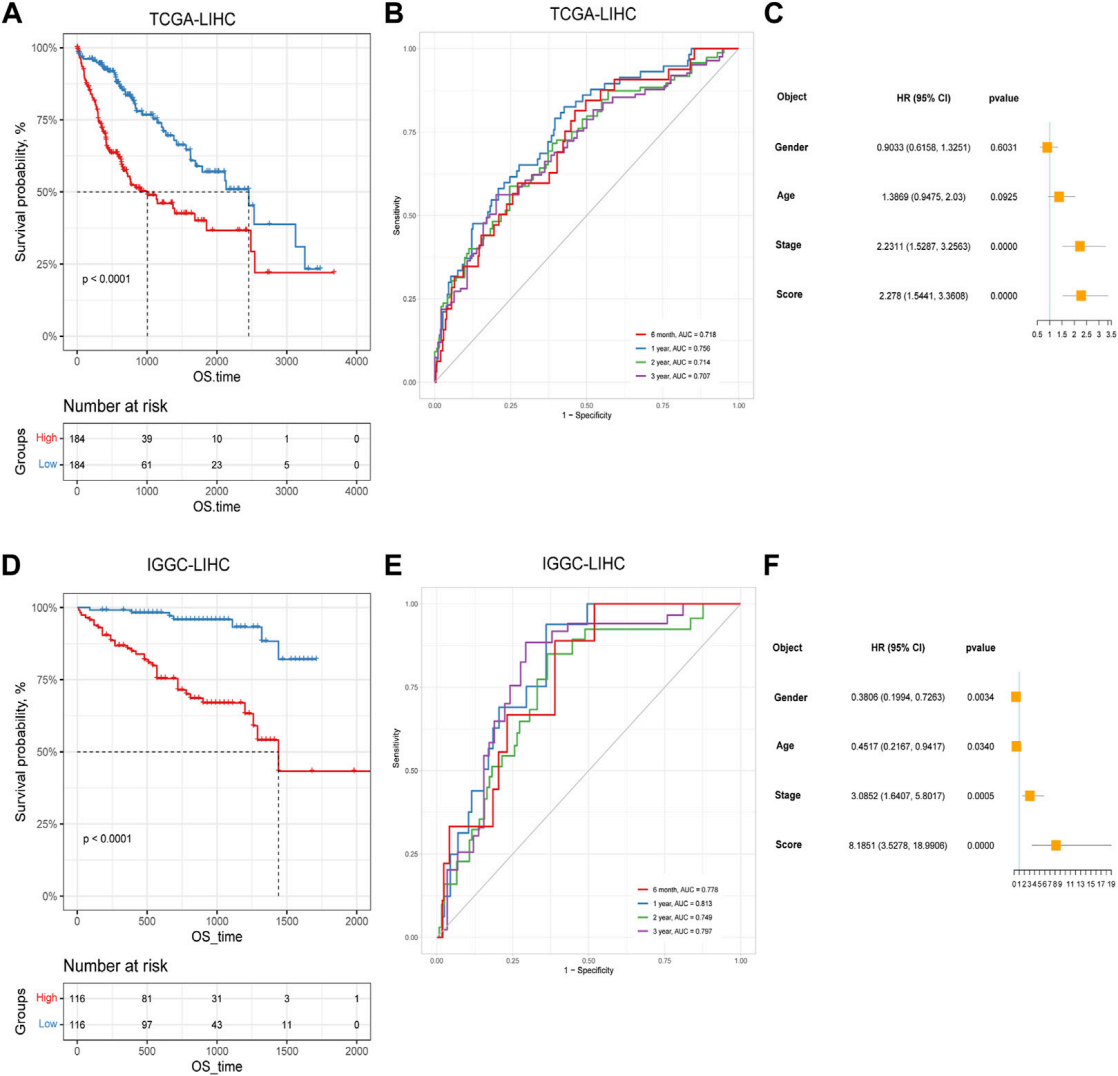
The prognostic effect of cuproptosis-related genes signatures in hepatocellular carcinoma. **(A)** Survival analysis of patients in TCGA cohort based on cuproptosis score; **(B)** Time-dependent receiver operating characteristic (ROC) curve of cuproptosis score in TCGA dataset; **(C)** Multivariate Cox analysis results in TCGA dataset; **(D)** Survival analysis of patients in LIRI-JP cohort based on cuproptosis score; **(E)** ROC curve of cuproptosis score in the LIRI-JP dataset; **(F)** Multivariate Cox analysis results in LIRI-JP dataset.

Based on the identified two subtypes of CRGs in HCC, we analyzed the differentially expressed genes (1,984 genes downregulated and 547 genes upregulated) between the two subtypes. We further performed GO and KEGG enrichment analysis for the differential genes, which were mainly enriched in pathways involved in cell proliferation (organelle fusion, nuclear division, etc.) and cell communication (neuroactive ligand, receptor interaction, etc.) (Figures 5A, B). Then, we performed LASSO and multivariate COX analysis on the two subtypes of differential genes, and obtained a five-gene signature model (*G6PD*, *PRR11*, *KIF20A*, *EZH2*, and *CDCA85*) (Figures 5C–E). The Kaplan-Meier analysis revealed that the CRGs signature was associated with patients’ prognosis, and the patients in the high-risk group had an inferior overall survival (OS, *p* < 0.0001, Figure 6A). We further performed the time-dependent receiver operating characteristic (ROC) curve with the area under the curve (AUC). The AUC values of 6 months, 1-, 3-, and 5-year survival rates of prognostic subgroups were 0.718, 0.756, 0.714, and 0.707, respectively (Figure 6B). Meanwhile, we further validated the prognostic performance of the CRGs model in the LIRI-JP dataset. Similarly, we gained parallel results in the validation set, indicating an excellent predictive prognostic accuracy of the CRGs model for HCC patients. The AUC values of 6 months, 1-, 3-, and 5-year survival rates of prognostic subgroups were 0.778, 0.813, 0.749, and 0.797, respectively (Figures 6D, E). In addition, multivariate Cox regression showcased that the CRGs signature was an independent risk factor for HCC in both cohorts (*p* < 0.0001, Figures 6C, F).

### 3.4 Analysis of CRGs signature with clinical characteristics

**FIGURE 7:**
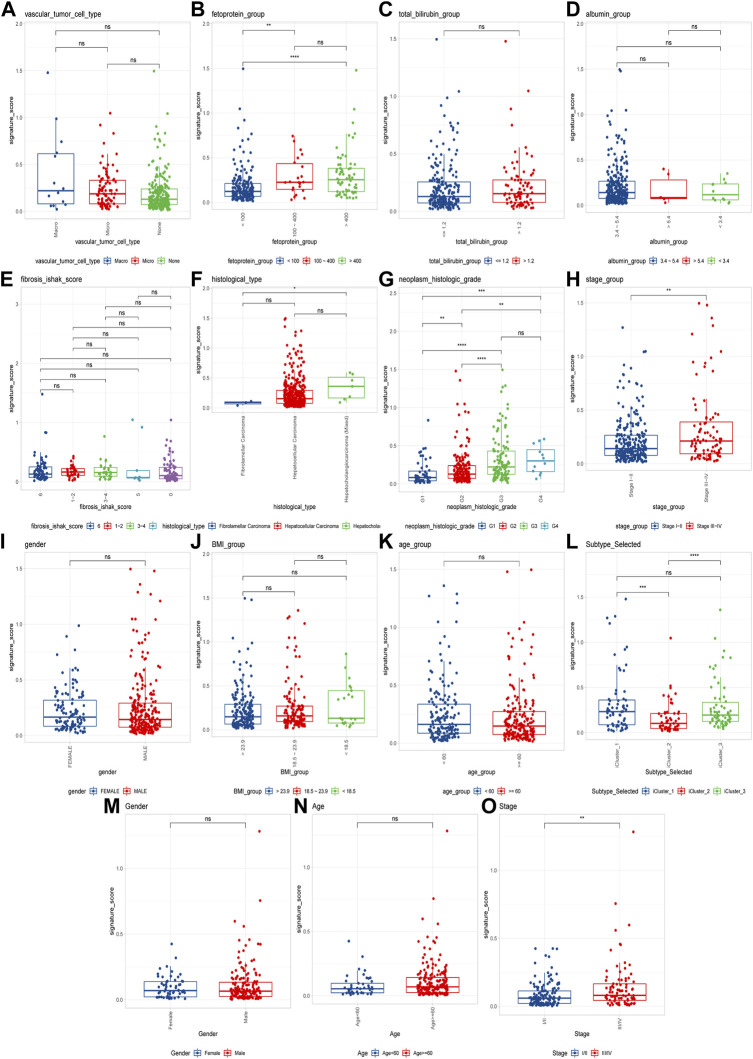
Cuproptosis-related genes signature correlates with clinical features. **(A–L)** Cuproptosis scores among different clinical features of hepatocellular carcinoma, including alpha-fetoprotein, total bilirubin, albumin, fibrosis score, histological type, histological grade, tumor stage, sex, BMI, age and molecular subtypes in TCGA cohort; **(M–O)** The differences of cuproptosis scores by gender, age, and tumor stage in the LIRI-JP cohort (ns: *p* >0.05; *: *p* <0.05; **: *p* <0.01; ***: *p* <0.001; ****: *p* <0.0001).

To explore the CRGs risk model with clinical characteristics, we found that the CRGs signature was associated with multiple clinical features, including alpha-fetoprotein, histological grade, tumor stage, as well as TCGA molecular subtypes, etc. (Figures 7A–N). In the LIRI-JP dataset, we verified that the CRGs signature significantly correlated to the tumor stage (Figure 7O).

### 3.5 Characterization of molecular landscape, immunotherapeutic and druggable responses of cuproptosis-related genes signature

We analyzed the CRGs’ genetic features based on the single nucleotide variants (SNV) and copy number variation (CNV) of HCC. We performed a chi-test in high and low CRGs groups with mutation frequency > 5%, and we observed no statistical significance between the two groups (Supplementary Figure S1A). We further characterized the high and low CRGs groups for copy number deletions and amplifications on chromosomes and also found no significant differences between the two groups (Supplementary Figure S1B). The GSVA enrichment analysis revealed that they differed significantly in the high and low CRGs groups, and the low CRGs group had more enriched pathways (Supplementary Figure S2).

**FIGURE 8:**
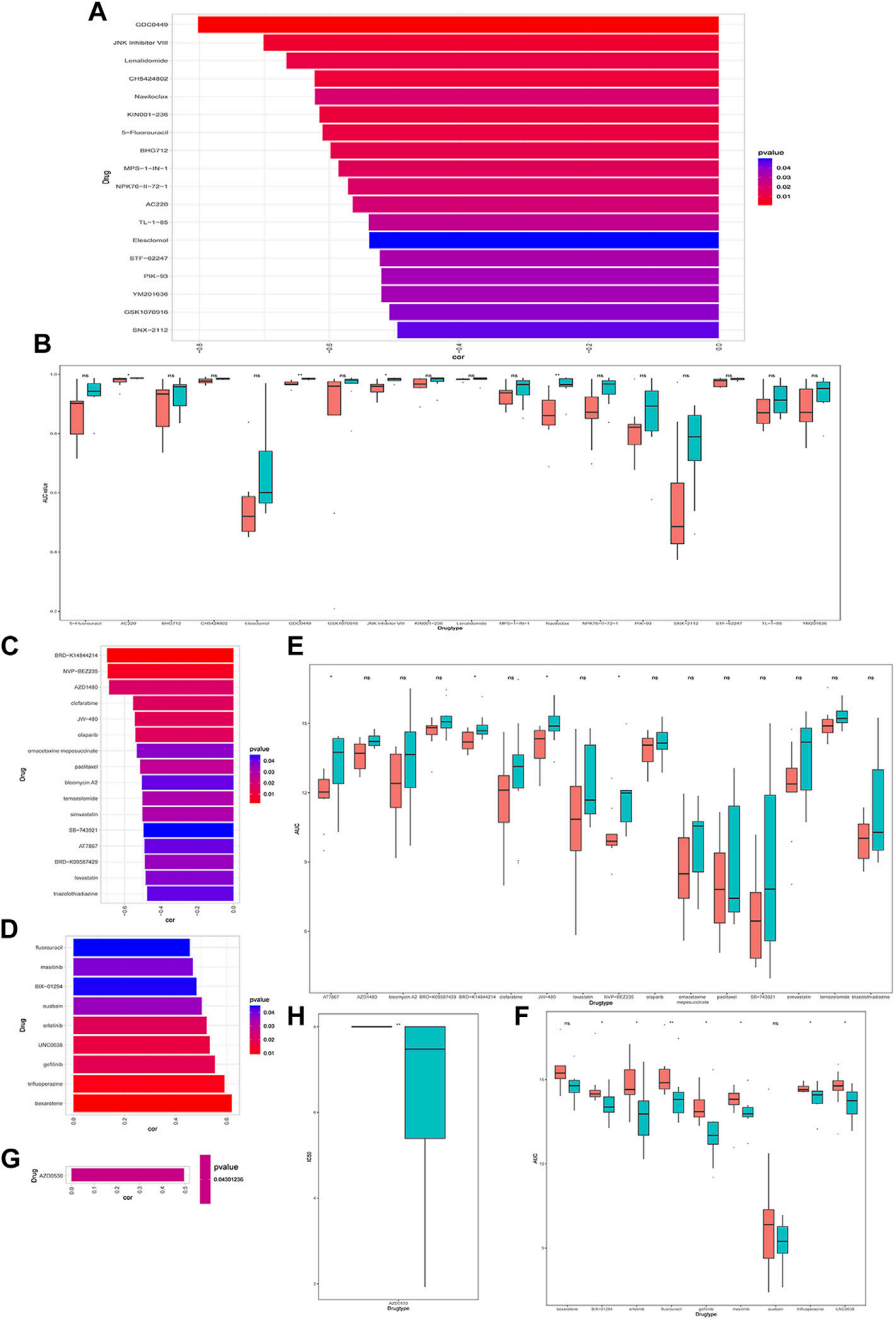
Drug sensitivity analysis of cuproptosis-related genes (CRGs)signature groups in Genomicsof Drug Sensitivity in Cancer (GDSC), Cancer Cell Line Encyclopedia (CCLE) and Cancer Therapeutics Response Portal (CTRP) databases. **(A)** Correlations between CRGs score and drug area under the curve (AUC) in GDSC (*p* < 0.05 was selected); **(B)** CRGs signature of each cell line under different drug treatments with significant negative correlation in GDSC database (* represents *p* <0.05, ** represents *p* <0.01, *** represents *p* <0.001). **(C)** Correlations between CRGssignature and drug AUC in CTRP database (*p* < 0.05 and drug display with negative correlation were selected). **(D)** Correlations between CRGs signature and drug AUC in CTRP database (select *p* < 0.05 and positive correlated drugs); **(E)** The CRGs signature of each cell line under different drug treatments with significant negative correlations in CTRP database (ns: *p* >0.05; *: *p* <0.05); **(F)** The differences in theCRGs signature of each cell line under different drug treatments with significant positive correlation in CTRP (ns: *p* >0.05; *: *p* <0.05; **: *p* <0.01); **(G)** The correlations between the CRGs signature in CCLE database and the IC50 of drugs (*p* < 0.05 and positive correlations were selected); **(H)** The differences in the CRGs signature of each cell line under different drug treatments with significant positive correlations in CCLE (*represents *p* <0.05; *represents *p* <0.01; **represents *p* <0.001).

Next, we explored the relationship between CRGs signature with drug sensitivity. We extracted cell line expression data based on GDSC, CCLE as well as CTRP databases, and combined them with the AUC/IC50 data for analysis. In the GDSC database, we discovered that the AUC was negatively correlated with CRGs signature for multiple drugs, such as 5-Fluorouracil, GDC0449 et al. (Figure 8A), and the AUC was significantly different between high and low CRGs groups (Figure 8B). We only discovered one drug AZD0530 with an IC50 positively correlated with CRGs signature in the CCLE database, and its IC50 was significantly different between high and low CRGs groups (Figures 8G, H). We also revealed that multiple drugs were associated with CRGs signatures in the CTRP database (Figures 8C–F).

**FIGURE 9:**
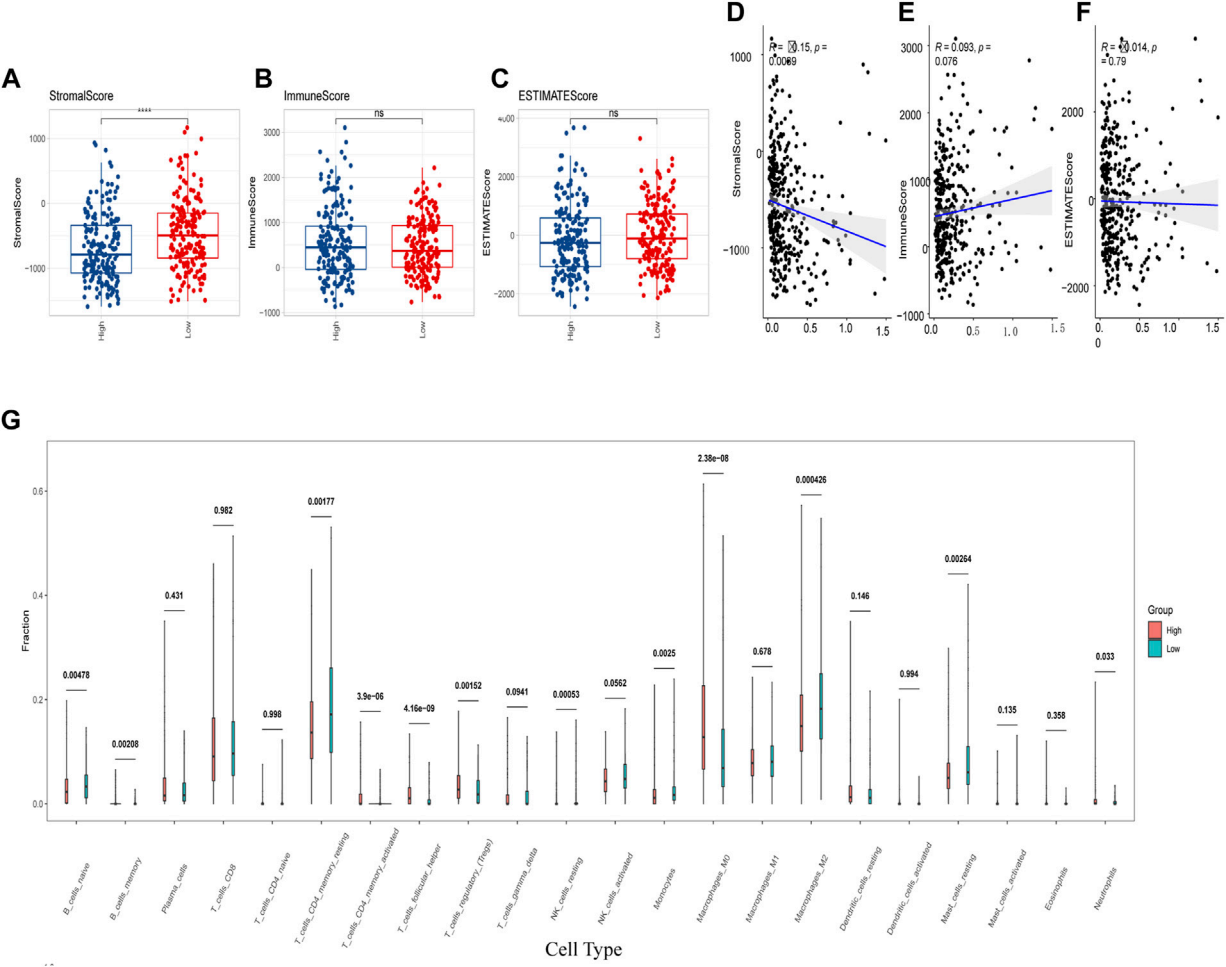
Cuproptosis-related genes (CRGs) signature was correlated with immune score and immune infiltrating cells. **(A–C)** Differences of matrix score **(A)**, immune score **(B)** and ESTIMATE score **(C)** between the two groups with high and low CRGs signature; **(D–F)** Correlations of CRGs signature and matrix score **(D)**, immune score **(E)** and ESTIMATE score **(F)**; **(G)** Differences in immune cell score between two CRGs groups with high and low CRGs signature (calculated by Cibersort) (ns: *p* >0.05; ****: *p* <0.0001).

**FIGURE 10:**
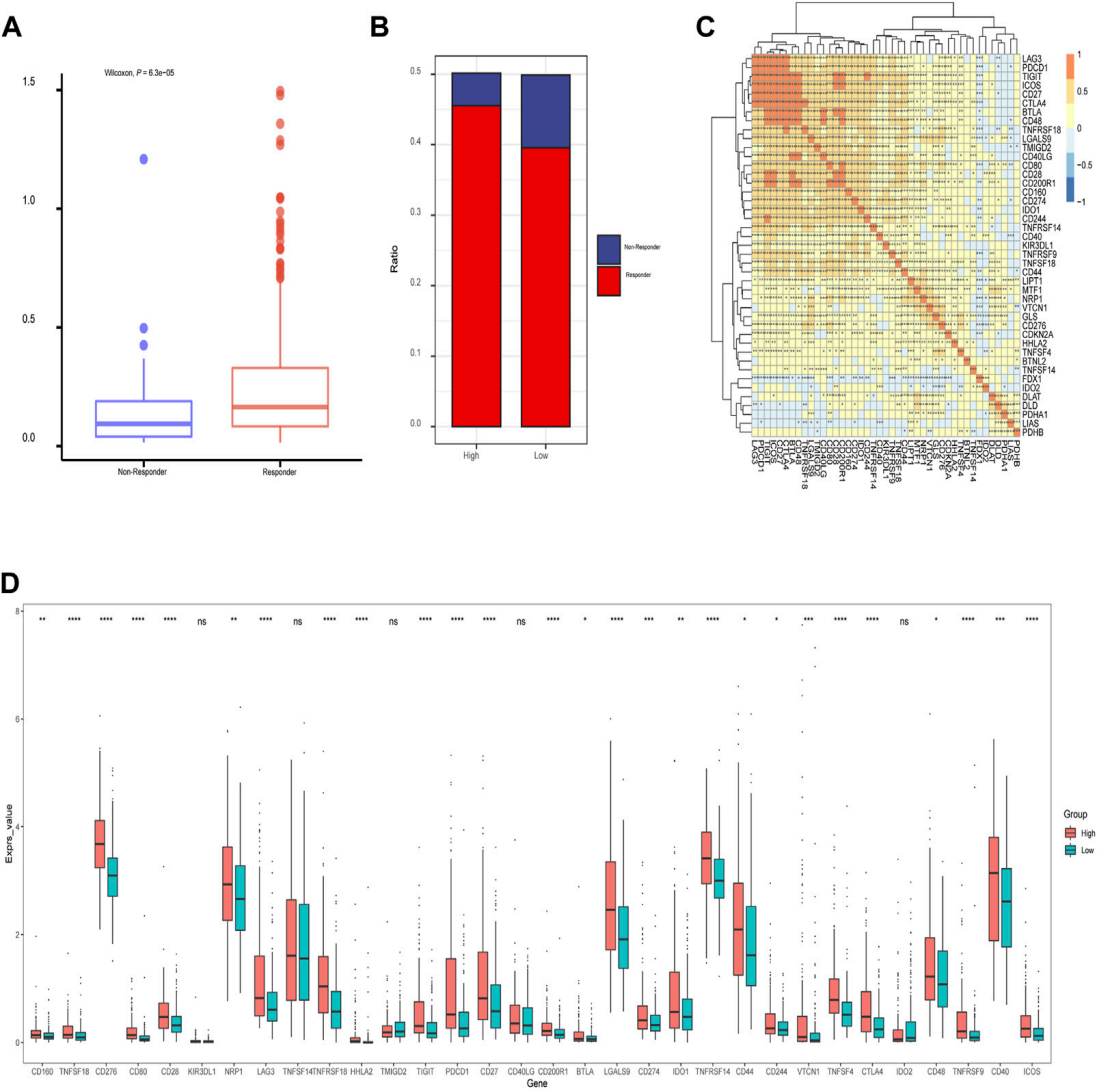
The relationship of immunotherapy responses and immune checkpoints in different cuproptosis-related genes (CRGs) signature groups. **(A)** Differences in CRGs signature of patients with different treatment outcomes (beneficial or non-beneficial) **(B)** the proportion of immunotherapy benefit and non-benefit between the two CRGs groups; **(C)** Heatmap of correlation analysis between CRGs groups and different immune checkpoints (*represents *p* <0.05; *represents *p* <0.01; **represents *p* <0.001; ***represents *p* <0.001); **(D)** Differences between the immune checkpoints and two CRGs groups (ns: *p* >0.05; *: *p* <0.05; **: *p* <0.01; ***: *p* <0.001; ****: *p* <0.0001).

In addition, we assessed the CRGs signature with the tumor microenvironment (TME). We evaluated the TME score, which included the stromal score, ESTIMATE score, and immune score between the two subtypes. We observed that there was no significant difference between the two groups in immune and ESTIMATE scores, while we discovered a higher stromal score in the low CRGs group (Figures 9A–C). The correlation analysis also revealed that the stromal score exhibited a significant negative correlation with the CRGs signature (Figures 9D–F). Moreover, multiple immune cell differences were differentially expressed between the two subtypes, such as T regulatory cells, macrophages, monocytes, etc. (Figure 9G). We then utilized TIDE for the immunotherapy response prediction, and we explored that the responders had higher CRGs scores and the high CRGs group also presented higher proportions of responding patients (Figures 10A, B). Furthermore, we found that there were significant correlations between immune checkpoints and CRGs (Figure 10C), and multiple immune checkpoints were differentially expressed between the two CRGs subgroups, such as CTLA4, LAG3, PDCD1 (PD-1), and CD274 (PD-L1) (Figure 10D), suggesting a potential role of the cuproptosis-related subtypes in immunotherapy.

## 4 Discussion

As one of the most severe malignancies in the world, current treatment strategies for HCC are rather limited (Llovet et al., 2016). In addition, the high heterogeneity of HCC and complicated risk factors make predicting prognosis much more difficult. Recent studies have shown that copper levels are significantly elevated in the serum and tumor tissue of cancer patients compared to healthy patients (Blockhuys et al., 2017)- (Ishida et al., 2013). Although dysregulation of copper homeostasis may trigger cytotoxicity, alterations in intracellular copper levels may affect cancer development and progression (Babak and Ahn, 2021). Recently, a new cell death pathway called cuproptosis has been noted, and it has been demonstrated that copper directly binds to lipoylated components of the tricarboxylic acid (TCA) cycle, leading to toxic protein stress, and ultimately cell death (Tsvetkov et al., 2022). Liver cirrhosis, one of the crucial causes of HCC, showed copper accumulation compared to a healthy liver (Poznanski et al., 2021). Recent evidence demonstrated that increased levels of redox-active free copper might be associated with acute hepatitis and, ultimately, HCC (Koizumi et al., 1998). The above evidence indicates that copper levels play a role in HCC, which suggests that cuproptosis may be closely related to liver malignancy, so it is vital to explore the significance of CRGs in the development and prognosis of HCC.

Cuproptosis genes are widely perturbed in HCC. First, based on TCGA transcriptome datasets, we found that the CRGs of HCC and normal tissues were differentially expressed, and *GLS* (glutaminase) and *CDKN2A* were found to be significantly upregulated in HCC. *GLS* has been reported to be associated with several cancers (Masisi et al., 2020)- (Matés et al., 2019). *CDKN2A* is a tumor suppressor gene on chromosome 9p21.3 that plays a role in tumor proliferation suppression (Zhao et al., 2016). However, *CDKN2A* is upregulated in HCC and strongly associated with inferior prognosis (Luo et al., 2021). These two cuproptosis genes may play a vital role in the development of HCC. Next, a PPI network was constructed with 10 cuproptosis genes, and after GO enrichment analysis, the associated genes were enriched in several pathways, including compound biosynthesis and energy metabolism, such as pyruvate acetyl CoA biosynthesis, tricarboxylic acid cycle, mitochondrial acetyl CoA biosynthesis and organic cyclic compound biosynthesis, etc., suggesting that cuproposis activity was associated with multiple cancer-related pathways. By calculating the expression correlations of 10 CRGs, we found that *LIAS*, *LIPT1*, *DLD*, *DLAT*, *PDHA1*, *PDHB*, *MTF1*, *GLS*, and *CDKN2A* showed a positive correlation with other genes in HCC, while *FDX1* negatively correlated with multiple genes. Similar results have been reported in other types of cancer, for example, *CDKN2A* is upregulated in endometrial cancer and may contribute to its pathogenesis (Su et al., 2015). *PDHA1*, *PDHB*, *DLAT* and *DLD* act synergistically in the pyruvate dehydrogenase complex deficiency (Inui et al., 2022). Zhang et al. combined with bioinformatics tools have analyzed the expression and prognostic significance of *FDX1*, a key regulator of copper-induced death in HCC (Zhang et al., 1994). However, the expression and function of other CRGs in HCC are poorly understood and need further exploration.

Subtypes identification of cuproptosis genes was analyzed based on TCGA HCC transcriptome data. The HCC patients can be divided into two subtypes, and there were obvious expression differences between *FDX1* and *LIPT1* in the two subtypes. It has been reported that *FDX1*, a key regulator of cuproptosis, is downregulated in HCC and its high expression is associated with inferior prognosis in HCC patients (Zhang et al., 1994). Recent evidence suggests that *LIPT1* is involved in the lipoic acid metabolic pathway (Chen et al., 2018). The lipoic acid moiety can be transferred from one protein to another, affecting the tricarboxylic acid cycle. *LIPT1* expression is elevated in melanoma biopsies, and is an independent favorable prognostic indicator in melanoma patients (Liu et al., 2018). When calculating immune cell infiltration scores between subtypes, we found that two groups (including *FDX1, LIPT1, DLAT, PDHA1, MTF1, GLS,* and *CDKN2A*) were significant diversity in different immune cells (including T cells, B cells, and macrophages), suggesting that is a possible predictive value for prognosis. *LIPT1* expression was positively correlated with *PD-L1* expression and negatively associated with Treg cell infiltration. Melanoma patients with high *LIPT1* expression had longer overall survival than those with low *LIPT1* expression after receiving immunotherapy, suggesting the predictive value of *LIPT1* for prognosis (Lv et al., 2021).

The differential genes of the two subtypes of cuproptosis in HCC were identified along with GO and KEGG enrichment analysis, which revealed enrichment mainly in pathways involved in cell proliferation and cell communication. Univariate Cox regression was performed to select genes with significant *p*-values (*p* <0.05), and five genes including G6PD, PRR11, KIF20A, EZH2, and CDCA8 were chosen to construct a cuproptosis-related signature after Lasso Cox regression. Glucose-6-phosphate dehydrogenase (G6PD) catalyzes a processive step in the oxidative pentose phosphate pathway to generate NADPH and nucleotide precursors, and G6PD depletion triggers TCA intermediates depletion. *In vivo*, G6PD impairment significantly inhibits KEAP1 mutant tumor growth (Ding et al., 2021). Additional studies have shown that G6PD promotes tumor growth by protecting cells from ROS (Hayes et al., 2020). PRR11 is a proline-rich protein that is encoded by the PRR11 gene. The PRR11 gene is located in the 17q23 amplified region. Copy regions of 17q23 are significantly enriched in brain tumors, lung, breast, and ovarian cancers (Zheng et al., 2017). The PRR11 is located in the 17q23 amplified region. Copy regions of 17q23 are significantly enriched in brain tumors, lung, breast, and ovarian cancers (Chen et al., 2015). It is highly expressed in malignant tumors, such as ovarian cancer and osteosarcoma tissues. Its expression level is associated with tumor size, Enneking stage, lymph node metastasis, and patient outcome (Li et al., 2021). Compared with normal hepatocytes, KIF20A expression was significantly upregulated in HCC HepG2 and Sk-hep1 cells, and silencing of KIF20A inhibited the proliferation of HCC cells and enhanced chemosensitivity and sorafenib sensitivity. Functional studies demonstrated that the knockout of KIF20A inhibited HCC cell proliferation (Wu et al., 2021). Upregulation of EZH2 expression in HCC is associated with unfavorable prognosis. The silence of EZH2 inhibits the HCC cell survival, migration and invasion, increased E-cadherin expression, and decreased N-cadherin and vimentin expression (Zhang et al., 2021). Cell division cycle associated 8 (CDCA8) is an essential component of the chromosome passenger complex (CPC). During mitosis, it is involved in the regulation of the dynamic localization of cells, and studies have suggested that CDCA8 can be used as a biomarker for the early diagnosis and prognosis prediction of HCC patients. In addition, CDCA8 may be an effective therapeutic target for HCC (Lv et al., 2021).

In addition, our study revealed diverse cuproptosis genes to be differentially expressed in distinct clinical features. For example, *GLS* was differentially expressed in distinct age stages, BMI groupings as well as different tumor stages, and *DLD* was differentially expressed in alpha-fetoprotein levels. There were also significant expression differences among TCGA molecular classification, bilirubin, albumin maximum, fibrosis, grade, gender, and other clinical subgroups along with the 10 cuproptosis genes. It could be found that cuproptosis genes presented expression differences among different clinical features, suggesting the involvement of cuproptosis-related genes in the prognosis and development of HCC. To further explore the potential mechanism and the role of CRGs’ prognostic value, we successfully established and validated CRGs signature, and analyzed the its prognostic values and clinical implications. Clustering and survival analysis by the median value of cuproptosis-associated signature revealed that signature was associated with HCC prognosis (*p* < 0.0001), and the areas under ROC curves were 0.718, 0.756, 0.714, 0.707 at 6 months, 1, 3, and 5 years, respectively. Meanwhile, we further validated its predictive accuracy in the LIRI-JP dataset of ICGC, and gained consistent results, and the areas under the ROC curves at 6 months, 1, 3, and 5 years were 0.778, 0.813, 0.749, 0.797, respectively. Survival analysis also suggested that the low CRGs signature was associated with a better prognosis (*p* < 0.0001). Besides, multivariate Cox regression showcased that the CRGs signature was an independent risk factor for HCC in both cohorts (*p* < 0.0001). Meanwhile, we revealed that significance was correlated with multiple clinical features including alpha-fetoprotein, histological grade, tumor stage as well as TCGA molecular subtypes in TCGA. In the LIRI-JP dataset, we verified that significance indeed showed a significant relationship with tumor stage. Therefore, our CRGs signature presented an excellent performance in predicting the prognosis of HCC patients, and provide new insights for the classification of HCC.

The hallmark enrichment score of tumors was calculated based on GSVA to evaluate the difference in hallmarks between the two CRGs groups, and we observed significant enrichments of multiple hallmark pathways in the low CRGs group compared with the high CRGs group. In the analysis of the tumor microenvironment, the stroma score in the high CRGs group was less significant than that in the low CRGs group, while the immune score and the estimated score were not statistically significant. Further analysis also revealed that the stromal score exhibited a significant negative correlation with the CRGs signature. The immune cell infiltration algorithm analysis also showcased multiple immune cell differences in the high and low CRGs groups. It has been reported that the expression of CDKN2A, GLS and LIPT1 is positively correlated with the abundance of CD8+T cells and neutrophils and CDKN2A expression positively correlated with the degree of tumor infiltration (Luo et al., 2021), which was in line with our study. It was also reported that in tumor-infiltrating cells, the levels of eosinophils, macrophages of M0 and M2 phenotypes, mast cell activation, and NK cell activation were positively correlated with the risk score in high-and low-risk groups (Li et al., 2022a). Additionally, we identified distinct immune checkpoint expression patterns in the two CRGs subgroups, which improved the effectiveness of immunotherapy in the era of personalized medicine in HCC.

Moreover, we further explored the drug sensitivity for the potential therapeutic possibilities of drugs in HCC. Our results showed that multiple drugs exhibiting a negative correlation between AUC and signature were found in the GDSC database, such as: 5-Fluorouracil, GDC044g et al., and the AUC was significantly different between high and low CRGs groups. Multiple drugs were also found to be correlated in the CTRP database, and the AUCs were obviously different between high and low CRGs groups. AZD0530, a drug with an IC50 positively correlated with CRGs signature in the CCLE, and its IC50 was significantly different between high and low CRGs groups.

In immune infiltration analysis, we observed a higher stromal score in the low CRGs group, the stromal score exhibited a significant negative correlation with the CRGs signature. Multiple immune cell differences were differentially expressed between the two CRGs subtypes, such as T regulatory cells, macrophages, monocytes, etc. We also discovered there was a significant association between immune checkpoints and CRGs, most notably *PDCD1* (PD-1), *TIGIT*, *CTLA4*, *ICOS*, *BTLA*, *CD28*, *LAG3*, and *CD27*. Previous studies demonstrated that the combination of immune checkpoint inhibitors (ICIs) and bevacizumab showed superiority over sorafenib in unresectable HCC (Finn et al., 2020)- (Ren et al., 2021), which was consistent with the mechanism of immune checkpoints. *PDCD1* (PD-1) was strongly associated with tumor mutation burden (TMB), microsatellite instability (MSI), and immune cell infiltration, and it can be used as a prognostic marker in several cancer types (Miao et al., 2020). *LAG3* was the most promising immune checkpoint after PD-1 and CTLA-4, and higher *LAG3* and *FGL1* expression promoted tumor growth by suppressing the immune microenvironment (Shi et al., 2021). *CD27* played a critical role in T cell activation by providing costimulatory signals (Angelika and Anna, 2020). By grouping immunotherapy responses according to different CRGs signatures, we found that the high CRGs group had more sensitive to immunotherapy and presented higher proportions of responding patients. Besides, we found that multiple immune checkpoints were differentially expressed between the two CRGs subgroups, such as *CD276*, *CD80*, *CD28*, *CTLA4*, *LAG3*, *PDCD1* (PD-1), and *CD274* (PD-L1), indicating a potential role of the cuproptosis-related subtypes in immunotherapy. Zhou et al. (2022b) and Fu et al. (2022) also observed similar results to the current study, suggesting that CRGs were closely related to immune checkpoints. Bian et al., (Liao et al., 2022) also reported that in renal cancer, a prognostic risk score with CRGs expression signature exhibited good performance in predicting OS and PFS of patients and was significantly correlated with the level of immune infiltration and *PD-L1* expression, which was in consistent with our results. However, how cuproptosis or cuproptosis influencing drugs affect the function of anti-tumor immune cells remains unclear, and needs further exploration.

In the current work, we identified the signature of cuproptosis-related genes in HCC and developed a CRGs-based prognostic model, demonstrating a strong ability to predict the prognosis of HCC and assess treatment efficacy. Undoubtedly, our study still has certain shortcomings. Firstly, given the prognostic model was constructed and validated by utilizing data from public databases, further biological functional experiments were required to confirm our findings. Secondly, although a prognostic score focusing on the expression signatures of CRGs showed a favorable performance in predicting prognosis and clinical features in HCC, some vital clinical information was not available for analysis in the datasets, which would have impacted the prognosis and therapeutic effects of HCC. Finally, due to the limited sample size, a large-scale cohort study was crucial to evaluate the value of this model.

## 5 Conclusion

In summary, our integrative analysis depicted a molecular profile of CRGs and demonstrated its clinical implications in HCC. By establishing a CRGs-based prognosis model with the five hallmark genes (*G6PD*, *PRR11*, *KIF20A*, *EZH2*, and *CDCA8*), it brought prospective targets for determining the therapeutic efficacy of immunotherapy and targeted therapy, and accurately predicting the survival of HCC. The model based on CRGs helped better guide risk stratification and treatment strategy for HCC patients.

## Data Availability

The datasets presented in this study can be found in online repositories. The names of the repository/repositories and accession number(s) can be found in the article/Supplementary Material.
