# Supplementary figures and images for: Identification and validation of a novel cuproptosis-related genes signature associated with prognosis, clinical implications and immunotherapy of hepatocellular carcinoma
Source: Front Pharmacol. 2023 Feb 9;14:1088993. doi: 10.3389/fphar.2023.1088993 (PMC9947158; doi:10.3389/fphar.2023.1088993)

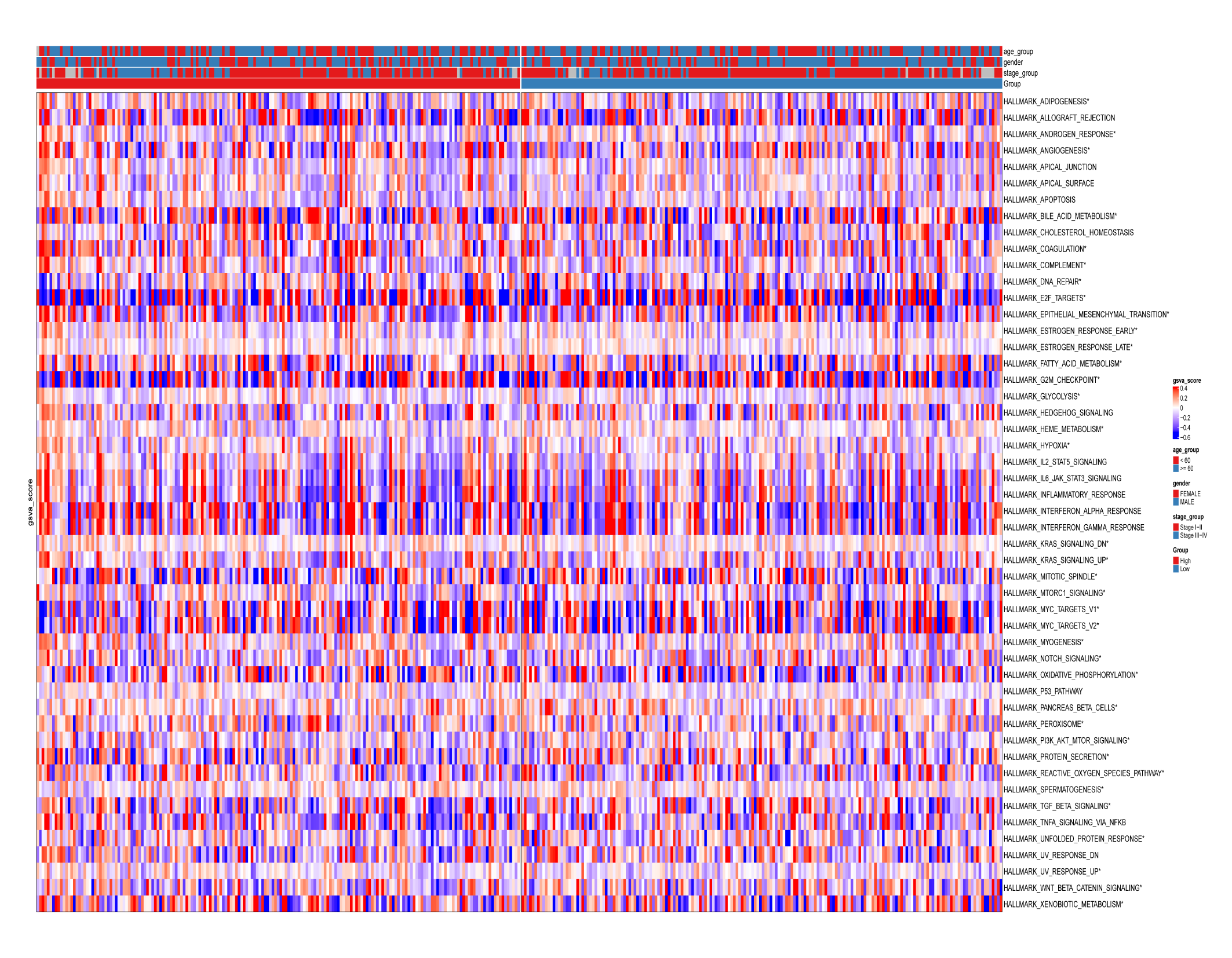

Supplement: Supplementary file 1 [file Image2.TIF]

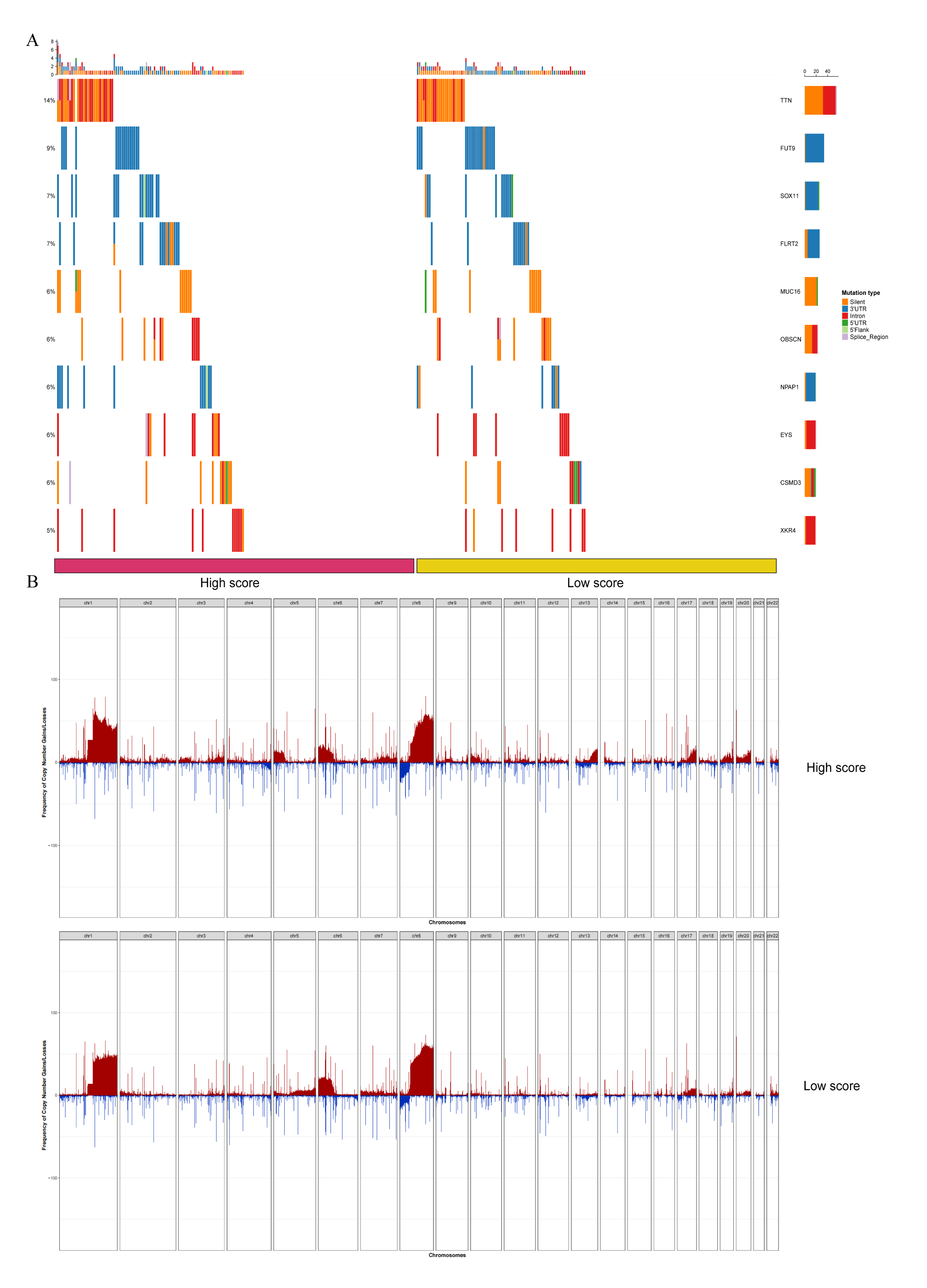

Supplement: Supplementary file 2 [file Image1.TIF]
